# The Partial Role of KLF4 and KLF5 in Gastrointestinal Tumors

**DOI:** 10.1155/2021/2425356

**Published:** 2021-07-27

**Authors:** Jun-Chen Li, Qiu-Han Chen, Rui Jian, Jiang-Rong Zhou, Yun Xu, Fang Lu, Jun-Qiao Li, Hao Zhang

**Affiliations:** ^1^Department of Gastroenterology, The Second Affiliated Hospital of Chongqing Medical University, Chongqing 400010, China; ^2^Blood Purification Center, University-Town Hospital of Chongqing Medical University, Chongqing 400000, China; ^3^Department of Gastroenterology, People's Hospital of Chongqing Banan District, Chongqing 401320, China; ^4^Department of Gastroenterology, Chongqing Sixth People's Hospital, Chongqing 400060, China; ^5^Department of Gastroenterology, Chongqing North-Kuanren General Hospital, Chongqing 401147, China

## Abstract

**Background:**

KLF4 and KLF5 are members of the KLF transcription factor family, which play an important role in many gastrointestinal tumors. To gain a deeper insight into its function and role, bioinformatics was used to analyze the function and role of KLF4 and KLF5 in gastrointestinal tumors.

**Methods:**

Data were collected from several online databases. Gene Expression Profiling Interactive Analysis (GEPIA), UALCAN database analysis, Kaplan-Meier Plotter analysis, LOGpc system, the Pathology Atlas, and the STRING website were used to analyze the data. We download relevant data from TCGA and then perform GO enrichment and KEGG enrichment analysis. The effects of KLF5 on gastric cancer cell proliferation were measured by CCK-8 assay. The effect of KLF5 on the expression of CyclinD1 and MMP9 was detected by Western blot.

**Results:**

KLF4 and KLF5 were differentially expressed in normal and tumor tissues of the gastrointestinal tract, and their differential expression is related to several genes or pathways. KEGG analysis showed that KLF5 was coexpressed with endocytosis-related genes. KLF5 promotes the proliferation of gastric cancer cells and the expression of metastasis-related molecules.

**Conclusion:**

KLF4 and KLF5 are of great significance for developing gastrointestinal tumors and can be used as therapeutic targets.

## 1. Introduction

Malignant tumors are common diseases that significantly threaten human health, and transcription factors play an important role in tumor occurrence and development. Krüppel-like factors (KLFs) and specific proteins (SPs) belong to the family of transcription factors, containing conserved zinc finger domains that bind to target DNA sequences [1]. The KLF/SP family is expressed in various tissues and has different tissue-specific activities and functions [2]. These genes not only regulate physiological processes such as embryogenesis, growth, development, differentiation, and proliferation but also regulate the pathogenesis of many diseases, including inflammatory diseases and cancers [3–6]. A previous study showed that the loss of KLF/SP transcription factors is associated with many human diseases and cancers [7]. In the digestive system, the KLF/SP transcription factor family members can regulate their homeostasis and pathophysiology. These transcription factors control multiple processes and are essential for the normal functioning of the digestive system [2].

Krüppel-like factor 4 (KLF4) belongs to the SP/KLF family, a transcription factor with a zinc finger structure [8]. KLF4 is a DNA-binding transcriptional regulator highly expressed in the skin and gastrointestinal tract epithelial cells, especially in cell differentiation regions [9]. However, KLF4 is downregulated in many patients with epithelial cancer, including esophageal cancer [10], gastric cancer [11], colorectal cancer [12], and bladder cancer [13], leading to cell proliferation. For example, the absence of KLF4 in the mouse esophagus can increase proliferation of epithelial cells [14]. KLF4 is often deleted in gastrointestinal tumors [15], which can cause tumor growth of gastric cancer and colorectal cancer [11].

Krüppel-like factor 5 (KLF5), also known as BTEB2 and IKLF, belongs to the KLF family [16]. KLF5 can regulate cell proliferation, cell cycle, apoptosis, and differentiation [9]. In addition, KLF5 may play an important role in various tumors, including breast cancer, prostate cancer, bladder cancer, skin cancer, colon cancer, and esophageal cancer [13, 17–19]. Studies have shown that there is a relationship between KLF5 and the occurrence and development of intestinal tumors. KLF5 is related to the mutation of APC and RAS genes. Both mutations are the two most common types of mutations in human colorectal cancer [20, 21]. In gastric cancer, the poorly differentiated subtype of gastric cancer shows high expression of KLF5 [22], and patients with high expression of KLF5 have a poor prognosis [23].

In gastrointestinal tumors, changes in the expression levels of KLF4 and KLF5 have an impact on tumor development [2]. Bioinformatics was used for analysis to understand the function and roles of KLF4 and KLF5 in gastrointestinal tumors.

## 2. Methods

### 2.1. GEPIA (Gene Expression Profiling Interactive Analysis)

The data of GEPIA comes from RNA sequencing expression data of 9,736 tumors and 8,587 normal samples of TCGA and GTEx projects. GEPIA uses standard processing pipelines to analyze data and is a newly developed interactive Web server. It can perform tumor/normal differential expression analysis, analysis based on cancer type or pathological stage, patient survival analysis, etc. [24].

### 2.2. UALCAN

UALCAN is a comprehensive and interactive Web resource for analyzing cancer OMICS data. UALCAN provides users with easy access to published cancer OMICS data (TCGA and MET500), patient survival analysis, gene promoter methylation analysis, gene correlation analysis, etc. [25].

### 2.3. KMplot (the Kaplan-Meier Plotter)

The Kaplan-Meier Plotter (http://www.kmplot.com/analysis/) is capable of accessing the effect of 54675 genes on survival in 21 cancer types. Sources of the system database include GEO, EGA, and TCGA. This tool can be applied to perform survival curve analysis [26].

### 2.4. LOGpc System (Long-Term Outcome and Gene Expression Profiling Database of Pan-Cancers)

The LOGpc system encompasses 209 expression datasets and provides 13 types of survival terms for 31310 patients of 27 distinct malignancies (http://bioinfo.henu.edu.cn/CRC/CRCList.jsp). This tool can be applied to perform survival curve analysis of ESCA [27].

### 2.5. TCGA (the Cancer Genome Atlas Database)

The relevant data of rectal adenocarcinoma were downloaded from the TCGA database (https://portal.gdc.cancer.gov/), and then, the R package was used for GO enrichment analysis and KEGG enrichment analysis.

### 2.6. STRING

The STRING (https://string-db.org/) database is a protein interaction (PPI network) database. STRING collects, scores, and integrates all publicly available sources of protein interaction information and supplements these sources by calculating predictions [28]. STRING allows users to visualize the subset as an interactive network and can also perform gene set enrichment analysis such as Gene Ontology and KEGG.

### 2.7. The Pathology Atlas

The Pathology Atlas contains mRNA and protein expression data from 17 different forms of human cancer. The resulting 5 million IHC cancer tissue images are presented here [29].

### 2.8. Cell Proliferation Assay and Western Blot

Cell proliferation rates were assessed by Cell Counting Kit 8 assay according to the supplier's instructions. The effects of KLF5 on gastric cancer cell proliferation were measured by CCK-8 assay. Total proteins were collected from gastric cancer cells using RIPA lysis and extraction buffer, and the concentration of proteins was measured using Bradford protein concentration assay kit. The effect of KLF5 on the expression of CyclinD1 and MMP9 was detected by Western blot (Supplemental 1).

### 2.9. Statistical Analysis

Data were presented as the mean ± SD, and the *t*-test was used to compare the two groups (using Prism 5 statistical software). *P* value less than 0.05 was considered statistically significant.

## 3. Results

### 3.1. KLF4 and KLF5 mRNA Expression Levels in Gastrointestinal Tumors

**Figure 1:**
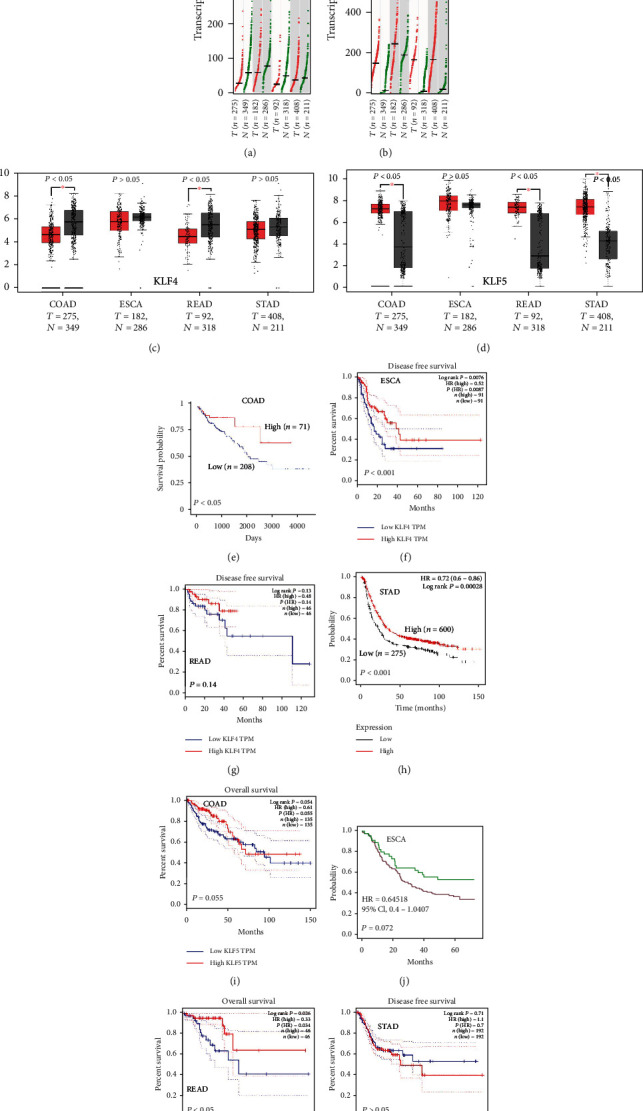
The expression and survival curve of KLF4 or KLF5 in gastrointestinal tumors. (a–d) Expression of KLF4 or KLF5 in gastrointestinal tumors. KLF4 was lowly expressed in COAD, ESCA, READ, and STAD tumors and had statistical significance in COAD and READ (*P* < 0.05). KLF5 was highly expressed in COAD, ESCA, READ, and STAD tumors and was statistically significant in COAD, READ, and STAD (*P* < 0.05). (e–h) The survival curve of KLF4 in gastrointestinal tumors. Patients with high expression of KLF4 had a good prognosis in COAD, ESCA, and STAD (*P* < 0.05). (i–l) The survival curve of KLF5 in gastrointestinal tumors. Patients with high KLF5 expression had a good prognosis in READ (*P* < 0.05). *T* means tumor and *N* means normal.

GEPIA was used to compare the expression levels of KLF4 and KLF5 between tumor tissues and normal tissues of the digestive tract (Figures 1(a)–1(d)). KLF4 was lowly expressed in gastrointestinal tumors, and the low expression in COAD and READ was statistically significant (*P* < 0.05) (Figure 1(c)). KLF5 is highly expressed in gastrointestinal tumors, and the low expression in COAD, READ, and STAD was statistically significant (*P* < 0.05) (Figure 1(d)).

### 3.2. Prognostic Analysis of KLF4 and KLF5 Expression and Patient Survival

To assess the correlation between KLF4 and KLF5 and clinical disease, UALCAN, GEPIA, and Kaplan-Meier Plotter were used for prognostic survival analysis. As shown in Figures 1(e)–1(h), the high expression of KLF4 in COAD, ESCA, and STAD showed a significant positive correlation with the survival rate of patients. As shown in Figures 1(i), 1(j), and 1(l), the survival rate of patients was poor with high expression of KLF5 in COAD, ESCA, and STAD. Although there was no significant difference (*P* > 0.05), we observed that the high expression of KLF5 was negatively correlated with the survival rate of patients according to the trend. Unlike above, the high expression of KLF5 in READ was significantly positively correlated with the survival rate of patients (*P* < 0.05) (Figure 1(k)).

### 3.3. Expression of KLF4 and KLF5 Based on Drinking Frequency or Helicobacter pylori Infection

**Figure 2:**
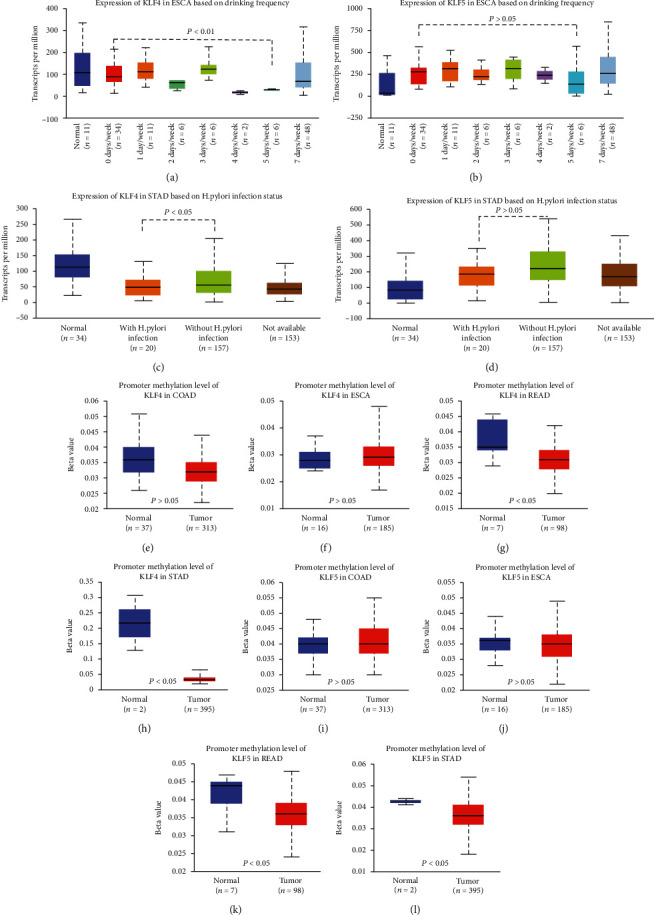
In ESCA and STAD, KLF4 expression is related to drinking frequency or Helicobacter pylori infection. The promoters of KLF4 and KLF5 genes are hypomethylated in READ and STAD. (a–d) Expression of KLF4 and KLF5 based on drinking frequency or Helicobacter pylori infection. (e–l) The methylation status of KLF4 and KLF5 promoters in gastrointestinal tumors.

UALCAN was used to compare the effects of different drinking frequencies for tumor patients on the expression levels of KLF4 and KLF5 in ESCA. In tumor patients with a history of drinking 5 days a week, the expression level of KLF4 in ESCA was significantly reduced (*P* < 0.01) (Figure 2(a)), whereas KLF5 did not show a significant difference (*P* > 0.05) (Figure 2(c)). Then, the relationship between KLF4 and KLF5 expression levels and Helicobacter pylori infection in gastric cancer was explored. We found that the expression level of KLF4 in STAD with Helicobacter pylori infection was significantly reduced (*P* < 0.05) (Figure 2(b)), while that of KLF5 was not significantly different in STAD with Helicobacter pylori infection (*P* > 0.05) (Figure 2(d)).

### 3.4. The Methylation Status of KLF4 and KLF5 Promoters in Gastrointestinal Tumors

UALCAN was adopted to analyze the changes in the methylation levels of KLF4 and KLF5 promoters between normal and tumor tissues of the gastrointestinal tract. We found that there was no significant change in the promoter methylation level of KLF4 in COAD and ESCA (*P* > 0.05) (Figures 2(e) and 2(f)), while the promoter methylation level of KLF4 was significantly reduced in READ and STAD (*P* < 0.05) (Figures 2(g) and 2(h)). The changes in the promoter methylation level of KLF5 in tumors were further analyzed, and no significant change was found in the promoter methylation level of KLF5 in COAD and ESCA (*P* > 0.05) (Figures 2(i) and 2(j)). However, in READ and STAD, the methylation level of KLF5 promoter was significantly reduced (*P* < 0.05) (Figures 2(k) and 2(l)).

### 3.5. PPI Network Analysis

**Figure 3:**
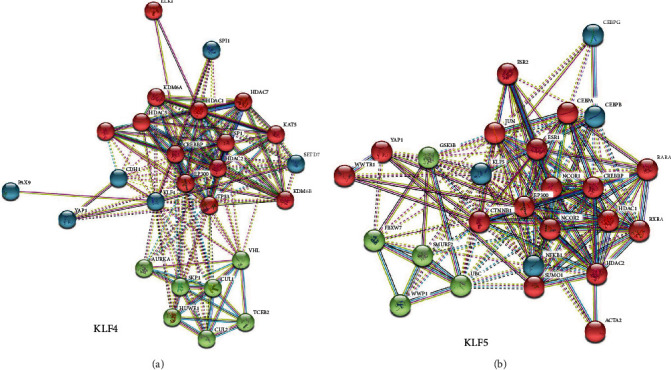
STRING interaction network of KLF4/KLF5 gene interacting proteins. (a) STRING interaction network of interacting proteins of KLF4 gene. (b) STRING interaction network of interacting proteins of KLF5 gene.

To identify protein molecules that interact with KLF4 and KLF5, the STRING database was used to generate a PPI network (Figure 3). The protein molecules (top 25) that interact with KLF4 include HDAC7, SP1, HDAC2, CTBP1, HDAC1, KDM6A, CREBBP, ELK1, KDM6B, EP300, HDAC5, KAT5, TP53, AURKA, CUL2, TCEB2, HUWE1, CUL1, SKP1, VHL, YAP1, SPI1, SETD7, CDH1, and PAX9 (Supplemental 2.3). The protein molecules (top 25) that interact with KLF5 include HDAC2, YAP1, EP300, CEBPA, CTNNB1, NCOR1, JUN, SUMO1, ACTA2, WWTR1, RARA, NCOR2, HDAC1, CREBBP, ESR2, RXRA, ESR1, FBXW7, UBC, SMURF2, GSK3B, WWP1, NFKB1, CEBPB, and CEBPG (Supplemental 2.4).

### 3.6. Gene Analysis of Coexpression with KLF4 or KLF5 in Rectal Adenocarcinoma (READ)

**Figure 4:**
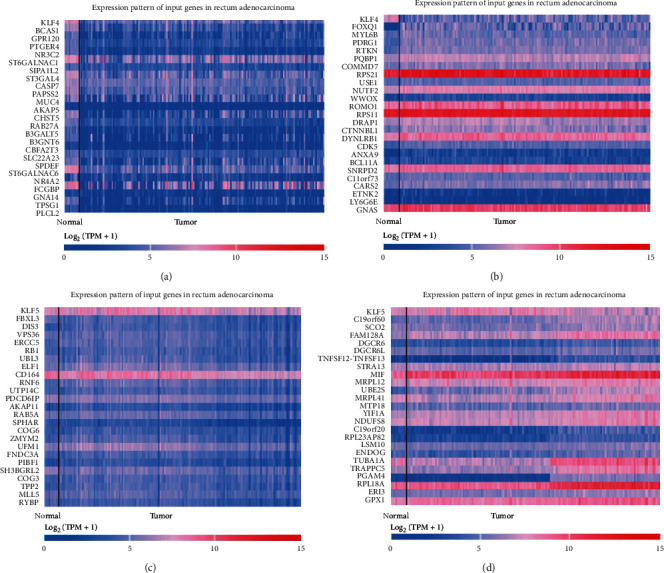
Genes related to KLF4/KLF5 in READ. (a) Genes positively correlated with KLF4 in READ. (b) Genes negatively correlated with KLF4 in READ. (c) Genes positively correlated with KLF5 in READ. (d) Genes negatively correlated with KLF5 in READ.

We used UALCAN to study which genes in rectal adenocarcinoma are related to the expression of KLF4 or KLF5. As shown in Figures 4(a) and 4(b), the genes positively or negatively related to KLF4 expression were analyzed, and the top 25 genes were listed. Then, the genes that were positively or negatively correlated with KLF5 expression were analyzed, and the top 25 genes were listed accordingly (Figures 4(c) and 4(d)).

### 3.7. GO Analysis and KEGG Enrichment Analysis of Genes Coexpressed with KLF5

**Figure 5:**
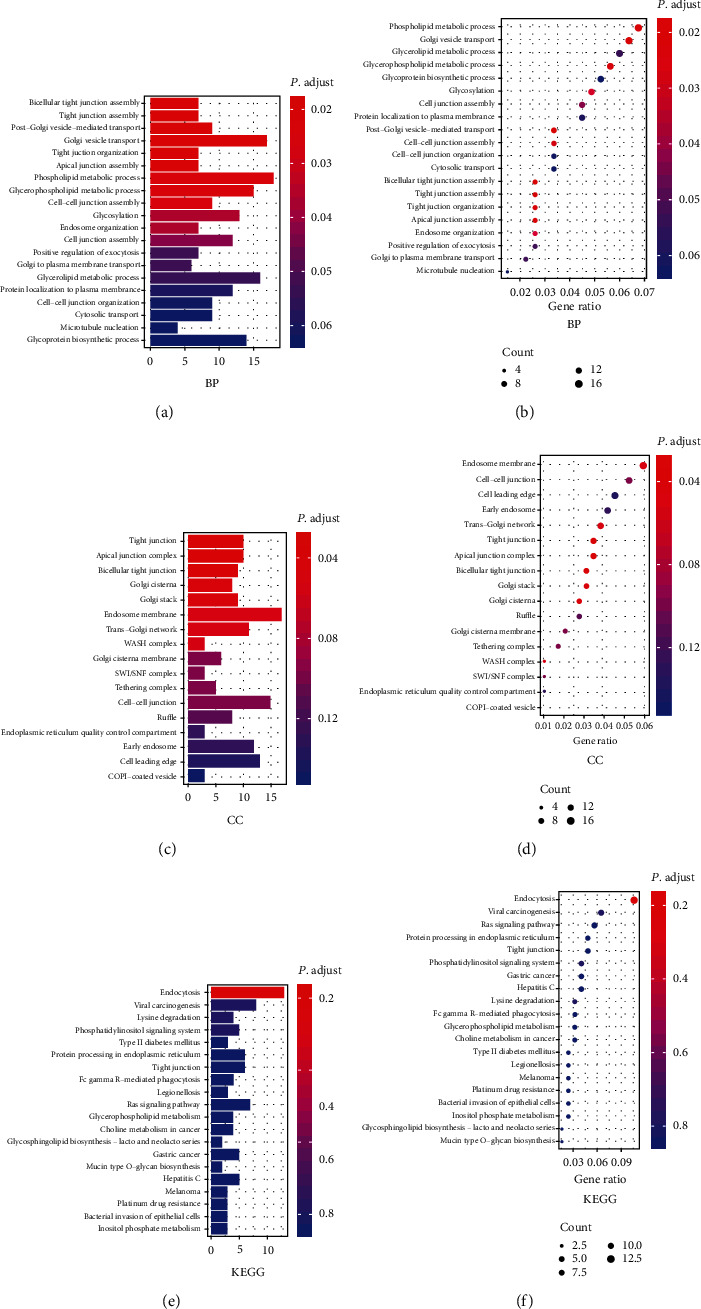
GO enrichment analysis of genes coexpressed with KLF5 in rectal adenocarcinoma. (a, b) Bar chart or bubble chart in biological process (BP) category. (c, d) Bar chart or bubble chart in cell component (CC) category. (e, f) KEGG enrichment analysis of genes coexpressed with KLF5 in rectal adenocarcinoma.

Taking KLF5 as an example, the related pathway of KLF5 coexpressed genes in rectal adenocarcinoma was analyzed. The data on genes coexpressed with KLF5 in rectal adenocarcinoma were downloaded and applied GO analysis on this data (Figure 5). The top 20 enriched GO terms are shown in Figures 5(a) and 5(b), including phospholipid metabolism process, Golgi vesicle transport, glycerolipid metabolism process, glycerophospholipid metabolism process, glycoprotein biosynthesis process, glycosylation, and cell junction assembly, in the biological process (BP) category. More genes were involved in phospholipid metabolism among these categories, and the metabolism was of great significance for tumor growth and metastasis.

The top 20 enriched GO terms in the cellular component (CC) category include endosome membrane, cell-cell junction, cell leading edge, early endosome, trans-Golgi network, tight junction, apical junction complex, and bicellular tight junction (Figures 5(c) and 5(d)). The cell connections involved in these categories were necessary for tumor metastasis.

The data of coexpressed genes with KLF5 were downloaded from TCGA, and then KEGG analysis was applied (Figures 5(e) and 5(f)). Genes coexpressed with KLF5 were enriched in endocytosis. Related literature has also shown that proper inhibition of endocytosis can sensitize tumor immunotherapy. Genes coexpressed with KLF5 were also abundant in the RAS pathway. Combined with relevant literature, the analysis illustrated that the expression of KLF5 was related to the regulation of these pathways.

### 3.8. KLF5 Promotes Proliferation of MGC-803 Cells

**Figure 6:**
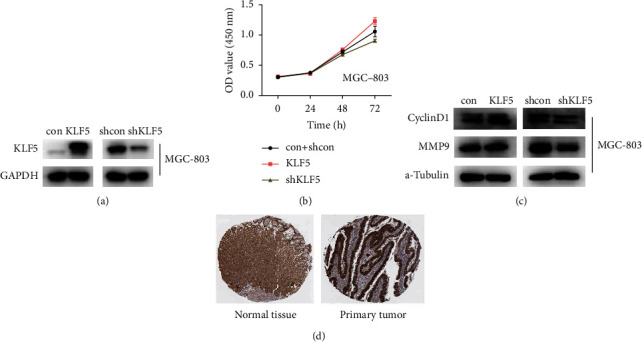
(a) MGC-803 cells were transfected with an overexpressing KLF5 plasmid to detect the expression levels of KLF5 protein. (b) CCK-8 detects the effect of KLF5 on cell proliferation. (c) MGC-803 cells were transfected with an overexpressing KLF5 plasmid and shKLF5 plasmid to detect the expression levels of CyclinD1 and MMP9 proteins. (d) Immunohistochemical analysis of KLF5 expression in stomach cancer and normal stomach tissue. Data derived from the Pathology Atlas database.

To verify the effect of KLF5 on MGC-803 cell proliferation, we tested whether shKLF5 inhibition can reduce the viability of MGC-803 cells. shKLF5 resulted in significantly decreased cell viability than the control group. Then, we tested whether overexpression of KLF5 promoted the viability of MGC-803 cells, and overexpression of KLF5 increased cell viability compared to the control group (Figure 6(b)).

### 3.9. KLF5 Regulates the Expression of Proliferating-Associated CyclinD1 and Metastasis-Associated MMP9 Proteins

To elucidate the mechanism by which KLF5 promotes proliferation and migration in gastric cancer, we next studied the expression levels of CyclinD1 and MMP9 proteins. We used MGC-803 cells, and CyclinD1 and MMP9 proteins showed a significant increase after overexpression of KLF5. Then, the inhibition of shKLF5 was tested. After treatment with shKLF5, CyclinD1 and MMP9 proteins were significantly decreased (Figure 6(c)).

## 4. Discussion

KLF4 and KLF5 are important members of the KLF family [7]. Due to their abundant expressions in gastrointestinal epithelial crypt cells were first identified as an intestinal-rich KLF (IKLF) [14, 30]. KLF4 and KLF5 have received extensive attention as a key transcription factor and potential drug target [19, 31].

In tumors, the deletion of a certain gene can lead to changes in the corresponding tumor suppressor function, thereby promoting tumors [32]. KLF4 exhibits cancer suppressive effects in gastrointestinal tumors, and its absence often leads to tumor deterioration [33]. Consistent with the previous reports, the results of GEPIA bioinformatics analysis exhibited that KLF4 showed low expression in gastrointestinal tumors. In addition, patients with high expression of KLF4 had a better survival rate. A previous study has shown that overexpression of KLF4 will lead to a decrease in the expression of N-cadherin, MMP2, and MMP9 [34]. On the contrary, the loss of KLF4 weakened the inhibition of N-cadherin, MMP2, and MMP9, leading to increased tumor cell invasion and migration, thereby affecting survival and prognosis [35]. KLF5, in contrast to KLF4, showed high expression in gastrointestinal tumors. High expression of KLF5 can promote cancer progression, such as the increase in KLF5 can promote breast cell proliferation and tumorigenesis [36]. Related literature indicates that patients with high expression of KLF5 have poor survival rates. However, our results indicated that patients with high expression of KLF5 in rectal adenocarcinoma have a higher survival rate. It may be caused by the difference in the sample size of the data.

P53 exerts its function and effect by regulating other genes [37]. When P53 is wild-type, KLF5 promotes the proliferation of esophageal keratinocytes. Conversely, when P53 is mutant, KLF5 inhibits cell proliferation [38]. However, whether P53 can affect the level of KLF5 protein remains to be elucidated. The presence of P53 can ensure the expression of KLF4 protein, and the tumor suppressor effect of KLF4 will be exerted [39]. A previous study has shown that KLF4 acts as a tumor suppressor in colorectal cancer and KLF4 plays an important role in *γ*-ray-induced DNA damage repair [40].

The direct contact of alcohol with the mucous membranes of the digestive tract can induce many changes in metabolism and function [41]. Excessive drinking not only can cause duodenal erosion, upper jejunal bleeding, and mucosal damage but also can regulate the intestinal mucosal immune system [42]. Alcohol-induced mucosal damage increases the risk of esophageal cancer [41]. We found that alcohol can affect the expression of KLF4 in esophageal cancer, and there is no significant difference in the effect of KLF5 expression. Among patients with esophageal cancer, those who had a history of drinking 5 days a week had lower KLF4 expression. Oxidative stress of alcohol can trigger chronic inflammation and carcinogenesis by forming reactive oxygen species [43]. Therefore, it was speculated that alcohol might affect the expression of KLF4 by causing certain metabolic changes.

Helicobacter pylori is a Gram-negative bacterium, which is the leading cause of chronic gastritis and peptic ulcer disease [44]. Helicobacter pylori's main virulence factor CagA can induce the occurrence of gastric cancer [45]. Meanwhile, CagA can upregulate miR-155 to inhibit the expression of KLF4 and promote the malignant transformation of normal epithelial cells [46]. It was revealed that gastric cancer patients infected by Helicobacter pylori had significantly lower KLF4 expression than noninfected patients. Therefore, Helicobacter pylori can play a role in promoting cancer by inhibiting the KLF4 expression.

Abnormal DNA methylation is relatively common in cancer, and most gene methylation abnormalities are prone to tumors [47]. In gastric cancer, abnormal DNA methylation in the gene promoter region can lead to the inactivation of tumor suppressor genes and other cancer-related genes and is an epigenetic marker in gastric cancer [48]. In colorectal cancer, DNA methylation can be used as a biomarker and evaluate its prognosis [49]. Bioinformatics analysis showed that in gastric cancer and rectal adenocarcinoma, the promoter region of KLF4 and KLF5 was significantly hypomethylated. It was speculated that in gastric cancer and rectal adenocarcinoma, abnormal methylation in the promoter region of KLF4 and KLF5 is involved in the progression or suppression of KLF4 and KLF5.

PPI plays an important role in various cellular pathways, allowing us to explore the function of proteins by understanding protein interaction partners [50]. The STRING database was used to analyze the proteins that interacted with KLF4 and KLF5. The pathways involved in the protein interaction between KLF4 and KLF5 are the Notch signaling pathway, general transcription pathway, and SUMO E3 ligase pathway. According to previous reports, the Notch signaling can enhance the expression of KLF4 and KLF5 and regulate the proliferation of conjunctival epithelium and goblet cell differentiation [51]. In intestinal tumors and colorectal cancer cells, the Notch signaling can inhibit the expression of KLF4 and reduce proliferation and tumor formation [52]. In bladder cancer, Notch-1 regulates the proliferation and differentiation of cancer cells by inhibiting the expression of KLF4 [53]. SUMOylation is a posttranslational modification that promotes nuclear localization of KLF5 by inhibiting NES activity [54]. It has been reported that SUMOylation is a key molecule that affects the function of KLF5 and transcriptional regulation that controls lipid metabolism [55]. The SUMOylation of KLF4 can regulate the transcription and proliferation of vascular smooth muscle cells [56]. By regulating the pathway of KLF4 and KLF5 interacting proteins, the expression of KLF4 and KLF5 can be regulated.

KLF4 plays an important role in stem cell renewal and reprogramming [57]. An important advantage of pluripotent stem cells (PSCs) is that they have the potential to proliferate indefinitely. However, this feature may also be a double-edged sword. The inherent tumorigenic properties, immunogenicity, and heterogeneity of stem cells are practical problems that limit the use of stem cells [58]. PSC KLF4 negatively regulates the epithelial cell-to-mesenchymal transition of gastrointestinal cancer by interacting with the Notch, TGF-*β*, and Wnt signaling pathways [15].

KLF5 was used as an example to analyze its coexpressed genes in colon adenocarcinoma. The abnormal lipid metabolism of cells has also received extensive attention and studied as potential pathogenesis of various tumors [59]. GO analysis showed that genes coexpressed with KLF5 are primarily involved in phospholipid metabolism in the biological process (BP) category. It has been reported that certain changes in lipid metabolism can promote tumorigenesis [60]. Changes in lipid metabolism can also activate important oncogenic signaling pathways, including the Hippo/YAP and Wnt/*β*-catenin pathways [61]. Interventions on key metabolites in KLF5 or its coexpressed genes and designing drug targets may be able to treat tumors.

GO analysis revealed that genes coexpressed with KLF5 are involved in cell junctions in the category of cell components. The cell connection is very important for tumor metastasis. If the tight connection of the cell is weakened, it will cause abnormal cell migration, thereby spreading the cancer cells [62]. Perhaps by regulating the expression of KLF5, it can reduce tumor metastasis and play an anticancer role.

KEGG analysis showed that genes coexpressed with KLF5 were enriched in endocytosis. The endocytosis mechanism regulates the interaction between cells and the environment by controlling the lipid and protein composition of the plasma membrane [63]. Endocytosis plays a vital role in cancer cell signal transduction, cancer cell invasion, and metastasis [64, 65]. It has been demonstrated that inhibition of endocytosis can effectively enhance the therapeutic effect of monoclonal antibodies against tumors and make tumor immunotherapy sensitive [66]. The coexpressed genes of KLF5 are enriched in endocytosis; thus, inhibiting or promoting the expression of these genes may regulate the expression of KLF5. Genes coexpressed with KLF5 are also abundant in the RAS pathway. RAS activation inhibits TGF-*β*-induced KLF5 acetylation [67], and the deacetylation state of KLF5 may be an important mechanism, by which KLF5 and HDAC promote cell proliferation and tumor growth [68]. Therefore, by regulating the expression level of KLF5 or its coexpressed genes, the development of cancer can be inhibited.

## 5. Conclusion

Through bioinformatics analysis, the functions and roles of KLF4 and KLF5 are further elucidated in gastrointestinal tumors. KLF4 and KLF5 may be used as potential therapeutic targets.

## Data Availability

The datasets generated and/or analyzed during the current study are available in the GEPIA, UALCAN, KMplot, LOGpc system, TCGA, and STRING repository: http://gepia.cancer-pku.cn/index.html, http://ualcan.path.uab.edu/index.html, http://kmplot.com/analysis/, http://bioinfo.henu.edu.cn/CRC/CRCList.jsp, https://portal.gdc.cancer.gov/, and https://string-db.org/.

## Abbreviations

TCGA: The Cancer Genome Atlas database
GEPIA: Gene Expression Profiling Interactive Analysis
COAD: Colon adenocarcinoma
ESCA: Esophageal carcinoma
READ: Rectum adenocarcinoma
STAD: Stomach adenocarcinoma
GO analysis: Gene Ontology analysis
BP: Biological process
CC: Cell components
KEGG: Kyoto Encyclopedia of Genes and Genomes.

## Supplementary Materials

Supplementary Materials Supplemental 1: cell proliferation assay and Western blot method. Supplemental 2: reaction pathways involved in KLF4 or KLF5 interacting proteins (top 6). The top 25 genes in KLF4's or KLF5's PPI network. Supplemental 3: the expression levels of KLF4 and KLF5 at various pathological stages. Expression of KLF4 and KLF5 in gastrointestinal tumors based on patient gender.
