# Supplementary material for: The Partial Role of KLF4 and KLF5 in Gastrointestinal Tumors
Source: Gastroenterol Res Pract. 2021 Jul 27;2021:2425356. doi: 10.1155/2021/2425356 (PMC8337138; doi:10.1155/2021/2425356)
Supplement: Supplementary Materials — Supplemental 1: cell proliferation assay and Western blot method. Supplemental 2: reaction pathways involved in KLF4 or KLF5 interacting proteins (top 6). The top 25 genes in KLF4's or KLF5's PPI network. Supplemental 3: the expression levels of KLF4 and KLF5 at various pathological stages. Expression of KLF4 and KLF5 in gastrointestinal tumors based on patient gender. [file 2425356.f1.zip › Supplemental 1.docx]

**Cell culture**

Human gastric cancer cells MGC-803 cell lines (laboratory preservation) were cultured at 37°C and 5% CO_2_ with high glucose Dulbecco's modified Eagle medium (HyClone, USA) supplemented with 10% FBS (lonser, Uruguayan) and 1% penicillin/streptomycin (Beyotime Biotechnology, Shanghai, China).

**Cell transfection**

Cells were plated in a 6‐well plate and grown to conﬂuence of 70-90% without antibiotics. Transfections were performed with Lipofectamine 2000 (Thermo Fisher Scientific), according to the manufacturer's instructions. The Plasmid and Lipofectamine 2000 were added into Eppendorf tubes containing 70µL OPTI-MEM. Following, the mixture was added to well after incubating for 15 minutes at room temperature. The medium was changed after 6 hours. Following transfection for 48 hours, the cells were collected to detect the expressions of related molecules. KLF5 was purchased from MiaoLingBio (Wuhan Miaoling Bioscience & Technology Co., Ltd., China.), ShKLF5 and negative control (shcon) were purchased from genechem (Shanghai Genechem Co., Ltd., China.).

**Cell proliferation assay**

Cell proliferation rates were assessed by Cell Counting Kit 8 assay (CCK-8, Beyotime, China) according to the supplier's instructions. Differently treated cells (3x 10^3^) were inoculated into 96-well plates containing 100μl of DMEM medium per well. 10μL CCK-8 is added to each well at the specified time point (0 hours, 24 hours, 48 hours and 72 hours) and incubation was incubated at 37°C for 1-2 hours. Absorbance of each Wells were measured at 450 nm using an ELISA plate reader.

**Western blot**

Total proteins were collected from gastric cancer cells using RIPA lysis and extraction buffer (Beyotime Biotechnology, Shanghai, China), and the concentration of proteins was measured using Bradford protein concentration assay kit (Beyotime Biotechnology, Shanghai, China). Then, proteins were separated by 10% or 12% SDS-PAGE and transferred to PVDF membranes. The membranes were blocked with 5% non-fat milk and incubated with primary antibodies against MMP9 (1:1000; #3852, Cell Signaling Technology, USA), KLF5 (1:1000; sc-398470, Santa Cruz Biotechnology, USA), CyclinD1 (1:1000; sc-8396, Santa Cruz Biotechnology, USA). GAPDH (1:1000; KC-5G4, Shanghai Kangchen), a-Tubulin (1:1000; AF0001, Beyotime Biotechnology, Shanghai, China) overnight at 4°C. Subsequently, the membranes were incubated with HRP-conjugated secondary antibody for 1 hour. The bands were visualized using enhanced chemiluminescence (ECL) kit (Thermo Fisher Scientific) and recorded with software.
