# Supplementary material for: The Partial Role of KLF4 and KLF5 in Gastrointestinal Tumors
Source: Gastroenterol Res Pract. 2021 Jul 27;2021:2425356. doi: 10.1155/2021/2425356 (PMC8337138; doi:10.1155/2021/2425356)
Supplement: Supplementary Materials — Supplemental 1: cell proliferation assay and Western blot method. Supplemental 2: reaction pathways involved in KLF4 or KLF5 interacting proteins (top 6). The top 25 genes in KLF4's or KLF5's PPI network. Supplemental 3: the expression levels of KLF4 and KLF5 at various pathological stages. Expression of KLF4 and KLF5 in gastrointestinal tumors based on patient gender. [file 2425356.f1.zip › Supplemental 2.docx]

**Supplemental 2**

**2.1** Table 1 Reaction pathways involved in KLF4 interacting proteins (top 6)

| **Term ID** | **Term description** | **False discovery rate (FDR)** |
| --- | --- | --- |
| HSA-2122947 | NOTCH1 Intracellular Domain Regulates Transcription | 8.42E-13 |
| HSA-2644606 | Constitutive Signaling by NOTCH1 PEST Domain Mutants | 2.37E-12 |
| HSA-2894862 | Constitutive Signaling by NOTCH1 HD+PEST Domain Mutants | 2.37E-12 |
| HSA-3108232 | SUMO E3 ligases SUMOylate target proteins | 1.79E-11 |
| HSA-157118 | Signaling by NOTCH | 1.20E-10 |
| HSA-212436 | Generic Transcription Pathway | 1.10E-08 |

**2.2**

| **Protein name** | **Protein description** | **Gene count** |
| --- | --- | --- |
| HDAC7 | Histone deacetylase 7 | 13 |
| SP1 | Transcription factor Sp1 | 13 |
| HDAC2 | Histone deacetylase 2 | 13 |
| CTBP1 | C-terminal-binding protein 1 | 13 |
| HDAC1 | Histone deacetylase 1 | 13 |
| KDM6A | Lysine-specific demethylase 6A | 13 |
| CREBBP | CREB-binding protein | 13 |
| ELK1 | ETS domain-containing protein Elk-1 | 13 |
| KDM6B | Lysine-specific demethylase 6B | 13 |
| EP300 | Histone acetyltransferase p300 | 13 |
| HDAC5 | Histone deacetylase 5 | 13 |
| KAT5 | Histone acetyltransferase KAT5 | 13 |
| TP53 | Cellular tumor antigen p53 | 13 |
| AURKA | Aurora kinase A | 7 |
| CUL2 | Cullin-2 | 7 |
| TCEB2 | Elongin-B | 7 |
| HUWE1 | E3 ubiquitin-protein ligase HUWE1 | 7 |
| CUL1 | Cullin-1 | 7 |
| SKP1 | S-phase kinase-associated protein 1 | 7 |
| VHL | Von Hippel-Lindau disease tumor suppressor | 7 |
| YAP1 | Transcriptional coactivator YAP1 | 6 |
| SPI1 | Transcription factor PU.1 | 6 |
| SETD7 | Histone-lysine N-methyltransferase SETD7 | 6 |
| CDH1 | Cadherin-1 | 6 |
| PAX9 | Paired box protein Pax-9 | 6 |

**2.3** Table 3 The top 25 genes in KLF4's PPI network

| **Protein name** | **Protein description** | **Gene count** |
| --- | --- | --- |
| HDAC2 | Histone deacetylase 2 | 17 |
| YAP1 | Transcriptional coactivator YAP1 | 17 |
| EP300 | Histone acetyltransferase p300 | 17 |
| CEBPA | CCAAT/enhancer-binding protein alpha | 17 |
| CTNNB1 | Catenin beta-1 | 17 |
| NCOR1 | Nuclear receptor corepressor 1 | 17 |
| JUN | Transcription factor AP-1 | 17 |
| SUMO1 | Small ubiquitin-related modifier 1 | 17 |
| ACTA2 | Actin, aortic smooth muscle | 17 |
| WWTR1 | WW domain-containing transcription regulator protein 1 | 17 |
| RARA | Retinoic acid receptor alpha | 17 |
| NCOR2 | Nuclear receptor corepressor 2 | 17 |
| HDAC1 | Histone deacetylase 1 | 17 |
| CREBBP | CREB-binding protein | 17 |
| ESR2 | Estrogen receptor beta | 17 |
| RXRA | Retinoic acid receptor RXR-alpha | 17 |
| ESR1 | Estrogen receptor | 17 |
| FBXW7 | F-box/WD repeat-containing protein 7 | 5 |
| UBC | Polyubiquitin-C | 5 |
| SMURF2 | E3 ubiquitin-protein ligase SMURF2 | 5 |
| GSK3B | Glycogen synthase kinase-3 beta | 5 |
| WWP1 | NEDD4-like E3 ubiquitin-protein ligase WWP1 | 5 |
| NFKB1 | Nuclear factor NF-kappa-B p105 subunit | 4 |
| CEBPB | CCAAT/enhancer-binding protein beta | 4 |
| CEBPG | CCAAT/enhancer-binding protein gamma | 4 |

**2.4** Table 4 The top 25 genes in KLF5's PPI network
