# Supplementary material for: The Partial Role of KLF4 and KLF5 in Gastrointestinal Tumors
Source: Gastroenterol Res Pract. 2021 Jul 27;2021:2425356. doi: 10.1155/2021/2425356 (PMC8337138; doi:10.1155/2021/2425356)
Supplement: Supplementary Materials — Supplemental 1: cell proliferation assay and Western blot method. Supplemental 2: reaction pathways involved in KLF4 or KLF5 interacting proteins (top 6). The top 25 genes in KLF4's or KLF5's PPI network. Supplemental 3: the expression levels of KLF4 and KLF5 at various pathological stages. Expression of KLF4 and KLF5 in gastrointestinal tumors based on patient gender. [file 2425356.f1.zip › Supplemental 3.docx]

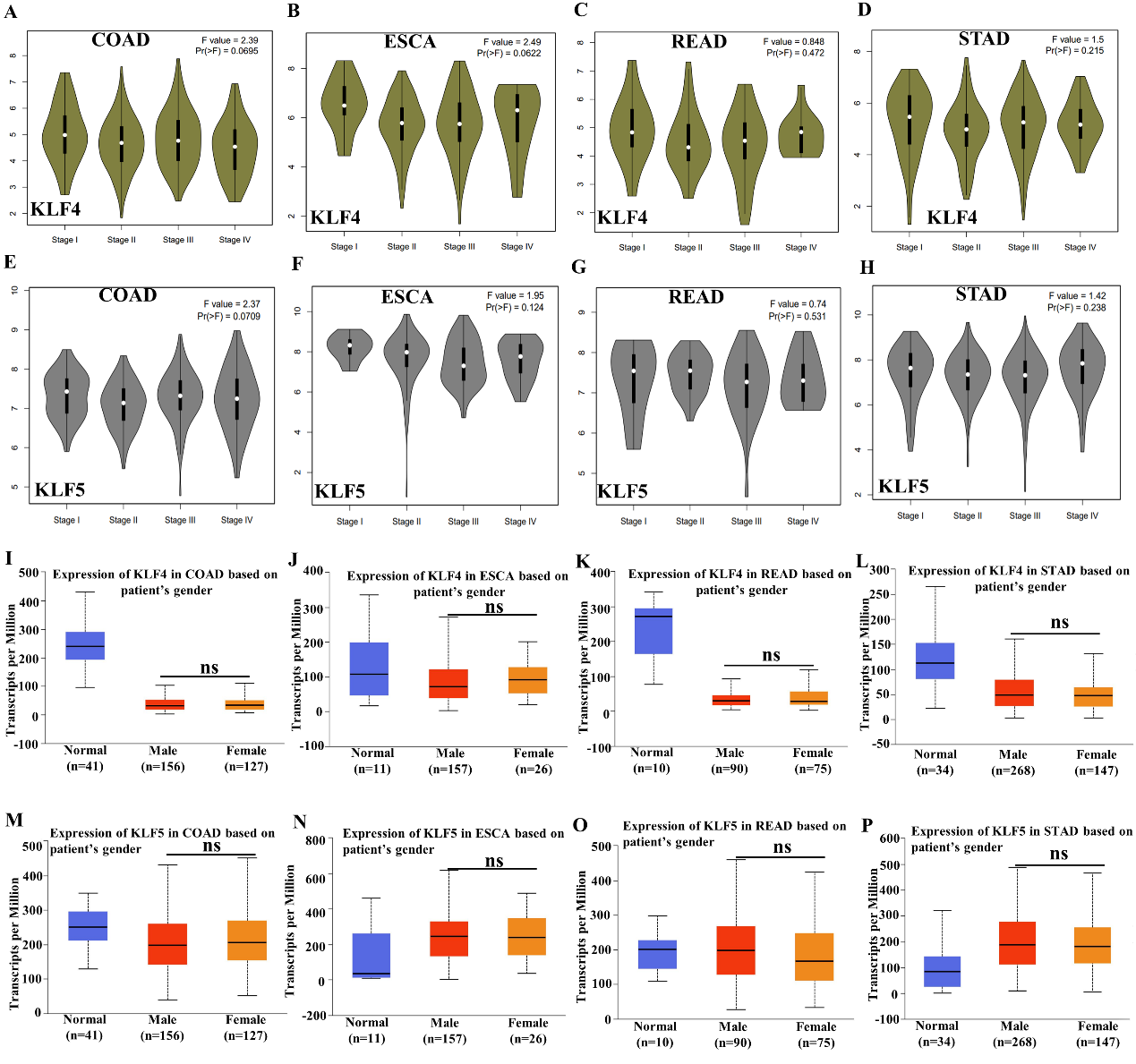


**Figure S.** In patients with gastrointestinal tumors, KLF4 / KLF5 expression has no relationship with tumor stage and gender. A-H. Correlation between KLF4/KLF5 expression and tumor stage in patients with gastrointestinal tumors (9,736 tumors and 8,587 normal samples). I-P. Expression of KLF4 and KLF5 in gastrointestinal tumors based on patient gender.

**Supplemental 3.1 The expression levels of KLF4 and KLF5 at various pathological stages**

GEPIA was used to compare the expression levels of KLF4 and KLF5 in various pathological stages of gastrointestinal tumor tissues. The expression levels of KLF4 in COAD, ESCA, READ and STAD did not change significantly at various pathological stages（Fig S A-D）. Then KLF5 was analyzed, revealing that its expression levels in COAD, ESCA, READ and STAD did not change significantly at each pathological stage（Fig S E-H）.

**Supplemental 3.2 Expression of KLF4 and KLF5 in gastrointestinal tumors based on patient gender**

UALCAN was applied to compare the expression levels of KLF4 and KLF5 in people with different genders in gastrointestinal tumors. For different genders, the expression level of KLF4 in COAD, ESCA, READ, STAD did not change significantly（Fig S I-L）. Then analysis on KLF5 showed that its expression level in COAD, ESCA, READ and STAD had no significant relationship with gender（Fig S M-P）.

Further bioinformatics analysis showed that the expression levels of KLF4 and KLF5 in gastrointestinal tumors at each pathological stage did not change significantly. Additionally, the expression of KLF4 and KLF5 had no significant difference in gender.
